# Influence of Ipsilateral Graft Inflow to Arteriovenous Fistula for Hemodialysis in Coronary Bypass Surgery

**DOI:** 10.3390/jcm11041053

**Published:** 2022-02-17

**Authors:** Bongyeon Sohn, Hyoung-Woo Chang, Jae-Hang Lee, Dongjung Kim, Junsung Kim, Cheong Lim, Kay-Hyun Park

**Affiliations:** 1 Department of Cardiothoracic Surgery, Bucheon Sejong Hospital, Bucheon-si 14754, Gyeonggi-do, Korea; scro@sejongh.co.kr; 2 Department of Thoracic and Cardiovascular Surgery, Seoul National University Bundang Hospital, Seoul National University College of Medicine, Seongnam-si 13620, Gyeonggi-do, Korea; changhw@snubh.org (H.-W.C.); truemed@snubh.org (J.-H.L.); mdknockout@snubh.org (D.K.); mluemoon@snubh.org (C.L.); 00913@snubh.org (K.-H.P.)

**Keywords:** coronary steal, steal phenomenon, coronary artery bypass grafting, arteriovenous fistula, myocardial ischemia

## Abstract

In coronary artery bypass grafting (CABG) for patients on hemodialysis, there has been concern about “coronary steal”. This study aims to evaluate the influence of using an in situ internal thoracic artery (ITA) ipsilateral to a preexisting arteriovenous fistula (AVF) in dialysis-dependent patients undergoing CABG. Between 2004 and 2018, dialysis-dependent patients with AVFs who underwent CABG were enrolled. According to the locational relationship of AVFs and in situ ITA grafts, the patients were divided into the ipsilateral group (*n* = 22) and the contralateral group (*n* = 21). Inverse probability weighting analysis was used to estimate and compare the late clinical outcomes. The late cardiac-related adverse events were not significantly different between the two groups: “major adverse cardiovascular and cerebrovascular events (MACCE)” (*p =* 0.090), “composite outcome of recurrent angina and coronary re-intervention” (*p* = 0.600). The in situ ITA graft of CABG on the ipsilateral side to AVF was not a significant risk factor for MACCE or the composite outcome of recurrent angina and coronary re-intervention. There was no statistically significant difference in the graft patency between the groups. Therefore, it might not be necessary to avoid using an in situ ITA on the ipsilateral side of an upper-arm AVF for optimal coronary artery bypass grafting in dialysis-dependent patients.

## 1. Introduction

In coronary artery bypass grafting (CABG) for patients dependent on hemodialysis, there has been concern about using an in situ internal thoracic artery (ITA) on the same side of an arteriovenous fistula (AVF). In particular, the issue of concern is a ‘steal’ phenomenon that can result from sharing a blood source between coronary perfusion and dialysis. Several studies have reported that angina could be aggravated during hemodialysis when an in situ ITA on the ipsilateral side of an AVF had been used for CABG [1,2,3]. Therefore, using an in situ ITA on the contralateral side of an AVF or performing aortocoronary bypass surgery with a saphenous vein graft is recommended. However, the benefits of using an ITA have been demonstrated in patients with renal insufficiency, with several reports about early graft failures in CABG using saphenous vein grafts [4]. Since an AVF is usually preferred on the patient’s non-dominant arm, a left-sided AVF is predominant [5]. Using an in situ right ITA can be considered an option in patients with an AVF on the left side. However, an important drawback to this option is that an in situ right ITA is frequently too short to reach the distal left anterior descending artery (LAD) by crossing the midline and this could result in a change in the whole graft configuration. There is a lack of related research and insufficient evidence to make optimal decisions in graft strategy. Therefore, this study aimed to evaluate the influence of using an in situ ITA graft ipsilateral to a preexisting AVF in dialysis-dependent patients undergoing CABG.

## 2. Patients and Methods

### 2.1. Patient Population and Inclusion Criteria

**Figure 1:**
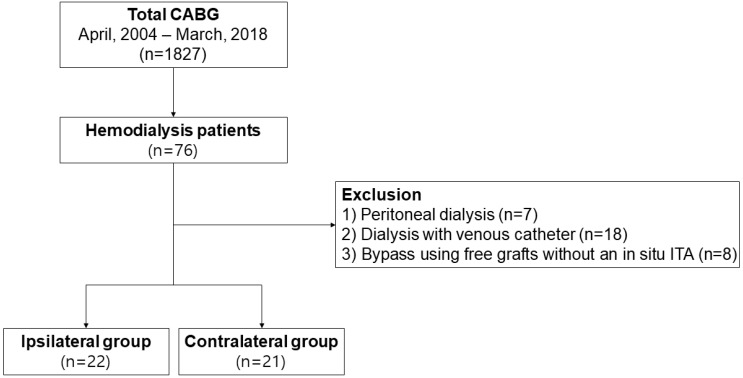
A diagram showing patient selection.

From April 2004 to March 2018, 1827 patients underwent CABG at our institution. We identified 76 patients with end-stage renal disease (ESRD) preoperatively requiring dialysis during the study period. Of them, 33 patients were excluded from the study: those who were dependent on peritoneal dialysis (*n* = 7), central venous catheter (*n* = 13), tunneled hemodialysis catheter (*n* = 5), and those who were operated upon using free grafts only without an in situ ITA (*n* = 8). Finally, 43 patients with an AVF who underwent CABG were enrolled in this study (Figure 1).

### 2.2. Surgical Procedures and Strategy

Both the internal thoracic and subclavian arteries were routinely evaluated preoperatively using computed tomography (CT) angiography. ITAs were harvested in a skeletonized fashion. Saphenous vein grafts (SVGs) were harvested using the “no-touch technique” by Souza et al. [6] The inflow selection was dependent on each surgeon’s preference. The graft selection and configuration strategies were as follows: an in situ left ITA was preferred for revascularization of the LAD territory whenever anatomically possible. In situ right ITAs were used for revascularizing the LAD by crossing the midline when necessary. Bilateral ITAs were primarily used as a composite graft. However, the use of bilateral ITAs was avoided in the dialysis-dependent patients with DM, owing to the concerns about postoperative sternal wound problems. SVGs were additionally used as second options of the composite graft from in situ ITAs or free grafts from the ascending aorta. The Y- or I-composite graft configuration was chosen by an operator in consideration of graft direction and number of anastomoses. Off-pump CABG was preferred, but cardiopulmonary bypass (CPB) was used when necessary. We routinely evaluated bypass graft flow using transit time flow measurement at the end of the operation for all patients.

### 2.3. Evaluation of Clinical Outcomes

The data of postoperative outcomes and events were acquired by reviewing the medical records or from direct telephone interviews with patients or their families or from the National Registry of Births and Deaths. The mean follow-up period was 3.9 ± 3.1 years. Early mortality was defined as any death within 30 days of surgery or during the same hospitalization period. Postoperative follow-up was performed regularly in an outpatient clinic with 3–12 month intervals. Coronary angiography or coronary CT was performed when angina symptoms developed. Clinical follow-ups were concluded on 31 March 2020. Major adverse cardiovascular and cerebrovascular events (MACCE) were defined as all-cause death, myocardial infarction, target vessel revascularization, and stroke. MACCE was reviewed as the primary endpoint of this study. We defined the secondary endpoint as the composite outcome of recurrent angina and coronary re-intervention for evaluating coronary steal.

### 2.4. Statistical Analysis

Statistical analyses were performed using IBM SPSS statistical software version 25.0 (IBM Inc., Armonk, NY, USA), STATA version 15.0 (StataCorp LP, College Station, TX, USA) and R software (version 3.4.0). The data are expressed as the means ± standard deviations, medians with interquartile ranges, or proportions. Comparisons of categorical and continuous variables were performed using the χ^2^ (or Fisher’s exact) test and Student’s *t*- (or Mann–Whitney U-) test, respectively. *p* < 0.05 was considered statistically significant. Time-related events were evaluated using the Kaplan–Meier curves and 2 groups were compared using the log-rank test. The risk factors were analyzed using the Cox proportional hazards ratio model. The variables with *p* < 0.10 were proposed for inclusion in the multivariable model.

### 2.5. Inverse Probability Weighting (IPW)

A total of 21 variables were used to estimate the patient’s case-weight. These variables were sex, age, body mass index, smoking, hypertension, diabetes mellitus, dyslipidemia, history of stroke, chronic obstructive pulmonary disease, atrial fibrillation, peripheral arterial occlusive disease, atherosclerosis in ascending aorta, acute myocardial infarction, history of old myocardial infarction, history of coronary intervention, ejection fraction, left ventricular dysfunction, three-vessel disease, left main disease, and emergency isolated coronary bypass surgery. A single propensity score was generated for each patient. With the estimated propensity score using the logistic regression model, a one-to-one matched cohort was constituted. The inverse probability treatment weights were calculated as follows: 1/(propensity score) for the group A and 1/(1 − propensity score) for the group B. The weights were normalized to 1. Weighted t-tests and weighted χ^2^ tests were used in the IPW-adjusted cohort to compare continuous or categorical variables in the 2 groups. A Cox proportional hazard regression model was adjusted for inverse probability weights.

## 3. Results

### 3.1. Baseline Characteristics

Before adjustment, the mean ages of the ipsilateral and contralateral groups were 63.0 ± 8.1 and 63.1 ± 9.1 years, respectively (*p* = 0.865). The proportion of three-vessel coronary disease was higher in the contralateral group (63.6% (ipsilateral) vs. 95.2% (contralateral), *p* = 0.021). The prevalence of peripheral arterial occlusive disease (PAOD) was greater in the contralateral group (18.2% (ipsilateral) vs. 47.6% (contralateral), *p* = 0.039) (Table 1*).* After IPW adjustment, no significant differences were observed between the groups regarding baseline parameters.

### 3.2. Operative Data

There was no statistically significant difference in the number of distal anastomoses between the groups (2.7 ± 1.4 (ipsilateral) vs. 3.1 ± 0.8 (contralateral), *p* = 0.361). The rate of complete revascularization was not different (68.2% (ipsilateral) vs. 76.2% (contralateral), *p* = 0.343). The use of Y-composite graft was similar in both groups (ipsilateral 45.5% vs. contralateral 33.3%, *p* = 0.537). Sequential anastomosis with I-composite graft was used in 1 patient (4.5%) of the ipsilateral group; however, 14 patients (66.7%) of the contralateral group had I-graft configuration. The LAD territory was revascularized in all the patients. However, the proportion of LAD anastomoses using in situ LITAs was significantly different: 21 (95.5%) patients in the ipsilateral group and 5 (23.8%) in the contralateral group (*p* ≤ 0.001). Concomitant surgery included mitral valve repair (*n* = 8), aortic valve replacement (*n* = 1) and double valve replacement (*n* = 1). The comparison of the operative characteristics between the two groups is shown in Table 2.

### 3.3. Early Outcome

Early outcomes were similar between two groups in an unmatched cohort. There were no significant differences between the two groups in immediate postoperative complications, including cerebrovascular accidents, arrhythmia, respiratory complication, wound infection, and bleeding reoperation. The early mortality rates for the ipsilateral and contralateral groups were zero (0%) and four patients (19%), respectively (*p* = 0.104). The causes of early death were cardiogenic shock with atrial fibrillation (*n* = 1), sepsis (*n* = 1), pneumonia (*n* = 1) and acute bowel ischemia (*n* = 1). After IPW-adjusting, early death rates were higher in contralateral group (*p* = 0.018) (Table 3). No patients had significant changes in chest pain or vital signs during dialysis before discharge.

### 3.4. Coronary Bypass Graft Patency Reports

Coronary angiography follow-ups were performed in 15 patients and coronary CT follow-ups were performed in the other 3 patients who were suspected of having recurrence of coronary disease. There was no statistically significant difference in the graft patency between the groups (75.9% ± 26.2% and 66.7% ± 37.3% in the ipsilateral and contralateral groups, respectively, *p* = 0.696).

### 3.5. Comparison of Five-Year Major Clinical Outcomes

**Figure 2:**
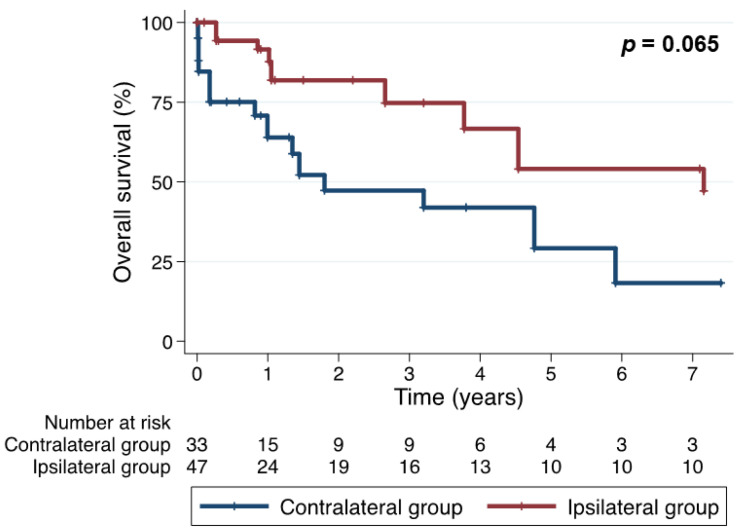
The overall survival comparison between the 2 groups (inverse probability weighting-adjusted comparison).

**Figure 3:**
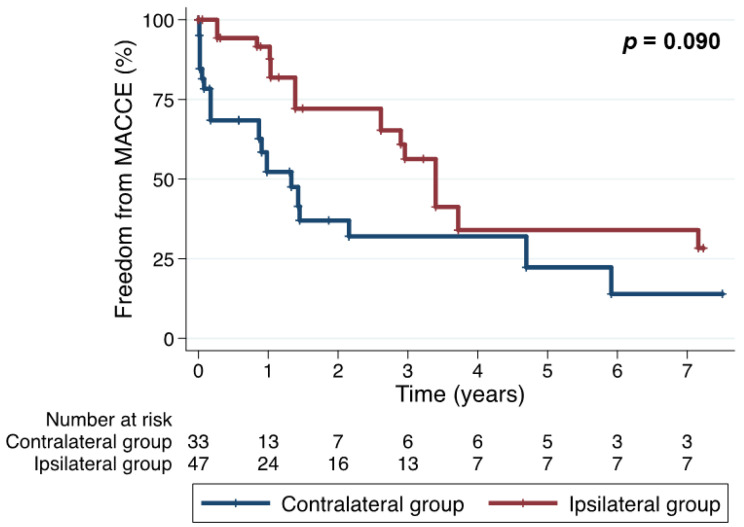
Freedom from major adverse cardiovascular and cerebrovascular events (inverse probability weighting-adjusted comparison).

**Figure 4:**
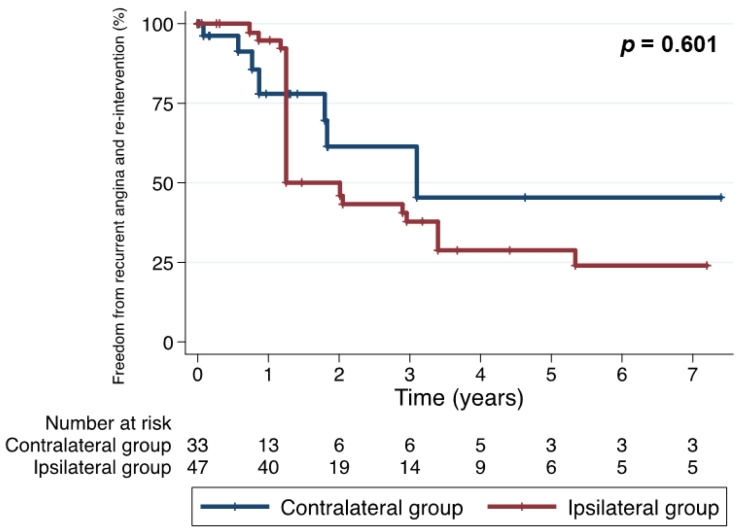
Freedom from the composite outcome of recurrent angina and coronary re-intervention (inverse probability weighting-adjusted comparison).

After IPW-adjusting, estimated major outcomes at 5 years were not significantly different between the two groups. The overall survival rates at 5 years of the ipsilateral and contralateral groups were 54.1% and 29.2%, respectively (*p* = 0.065) (Figure 2). Freedom from MACCE at 5 years were 33.9% and 22.3%, respectively (*p* = 0.090) (Figure 3). Freedom from the composite outcome of recurrent angina and coronary re-intervention at 5 years were 28.8% and 45.4%, respectively (*p* = 0.600) (Figure 4).

### 3.6. Risk Factor Analysis for Late Cardiac Events

#### 3.6.1. Before IPW-Adjustment

The multivariable Cox regression model revealed that using the in situ ITA on the ipsilateral side of the AVF was not a significant predictor of both MACCE (hazard ratio (HR), 0.495; 95% confidence interval (CI), 0.168–1.461; *p* = 0.203) and the composite outcome of recurrent angina and coronary re-intervention (HR, 0.635; 95% CI, 0.153–2.641; *p* = 0.533). Among the baseline characteristics, left ventricular dysfunction (HR, 5.180; 95% CI, 1.864–14.394; *p* = 0.002) and PAOD (HR 4.185, 95% CI, 1.349–12.979, *p* = 0.013) were independent risk factors for MACCE. Acute myocardial infarction and previous history of myocardial infarction were the independent risk factors of the composite outcome of recurrent angina and coronary re-intervention (HR, 3.705; *p* = 0.022 and HR, 3.617; *p* = 0.040, respectively). Of the operative parameters, I-graft configuration was associated with an increased risk of the composite outcome of recurrent angina and coronary re-intervention (HR 5.169, 95% CI, 1.342–19.907, *p* = 0.017) (Table 4).

#### 3.6.2. After IPW-Adjustment

Chronic obstructive pulmonary disease (HR, 5.667; 95% CI, 1.599–20.088; *p* = 0.007) and left ventricular dysfunction (HR, 4.016; 95% CI, 1.419–11.358; *p* = 0.009) were independent risk factors for MACCE. I-graft configuration was significantly associated with an increased risk of both MACCE (HR, 2.859; 95% CI, 1.080–7.565; *p* = 0.034) and the composite outcome of recurrent angina and coronary re-intervention (HR, 8.817; 95% CI, 1.859–41.809; *p* = 0.006) (Table 5).

## 4. Discussion

This study demonstrated two main findings. First, the ipsilateral group and contralateral group showed no significant difference in late cardiac events in terms of MACCE and the composite outcome of recurrent angina and coronary re-intervention. Second, the ipsilateral positioning of the in situ ITA graft to the AVF arm was not a significant risk factor of cardiac events after CABG. The “I-graft” configuration and preoperative patients’ characteristics, such as left ventricular dysfunction were more influential in late cardiac-related adverse events.

The prevalence of coronary artery disease in patients with hemodialysis has been described to be ~40% [7]. As the prevalence of ESRD continues to rise and the medical treatment for the population improves, the number of dialysis-dependent patients who require myocardial revascularization is expected to increase. Although renal insufficiency is associated with an increased risk of perioperative complications and mortality after CABG, surgical treatment showed favorable results compared with percutaneous coronary intervention [8,9]. In a study where 243 pairs of patients with ESRD on hemodialysis were analyzed, percutaneous coronary intervention was associated with a significantly higher risk of death and repeat revascularization, than CABG [10]. The freedom from revascularization at 5 years was higher in the CABG group than in the second-generation drug-eluting stent group in dialysis-dependent patients (93.4% vs. 79.1%, *p* = 0.013) [11]. Therefore, CABG is preferred in these patients. Since these patients have a higher restenosis rate than the non-hemodialysis patients, an appropriate surgical strategy is required [9,12].

One of the biggest concerns about CABG in hemodialysis-dependent patients is coronary steal phenomenon. There have been several reports on possible myocardial ischemia during hemodialysis owing to coronary steal in these patients. Gaudino et al. [3] reported hemodynamically evident flow-steal and myocardial ischemia during hemodialysis in patients with upper-extremity AVFs and an ipsilateral ITA to the coronary graft. In another study, significantly more cardiac events occurred in the group with an ipsilateral location of the ITA and AVF, than in the group with a contralateral location of the ITA and AVF [13]. Based on these findings, the clinical practice guidelines recommend avoiding the use of in situ ITA grafts on the ipsilateral side of an AVF [14]. However, several studies showed negative results regarding the use of ITAs on the ipsilateral or contralateral side of an AVF. One study showed similar overall survival (ipsilateral 58% vs. contralateral 65%) and cardiac event-free rates (ipsilateral 74% vs. contralateral 68%) at 5 years [15]. Feldman et al. [16] reported that there was no significant correlation between an in situ ITA-to-AVF relationship and the risk of cardiac events, as well as cardiac death. In situ ITA grafting ipsilateral to the AVF was not associated with early and 5-year worse outcomes. No patients developed coronary steal postoperatively [17].

This study demonstrated that the ipsilateral location of the in situ ITA graft was not a predictor of both MACCE and the composite outcome of recurrent angina and coronary re-intervention. This finding does not support the concern of coronary steal in dialysis-dependent patients associated with using an in situ ITA graft [3,13,14]. We tried to find another operative factor that may influence late cardiac events. One of the independent predictors of late cardiac events in our study was graft configuration. Since a left-arm AVF is predominant, the contralateral selection of the in situ ITA graft to the left AVF can have several disadvantages on graft strategy. For instance, although we prefer to revascularize the LAD with an in situ LITA, only 23.8% of patients in the contralateral group had distal anastomosis of the LAD territory with an in situ LITA from this study. RITA inflow graft was predominant at 76.2% of the contralateral group. For these patients, straight graft elongation using the I-graft configuration was necessary for achieving complete revascularization for three-vessel disease with the issue of graft length. The I-graft was used in 1 patient (4.5%) of the ipsilateral group, however, 14 patients (66.7%) of the contralateral group had I-graft configuration (*p* < 0.001). 

The Cox proportional hazards analysis revealed that the I-graft composite configuration was a significant risk factor of both MACCE and the composite outcome of recurrent angina and coronary re-intervention. I-graft is the elongation of the in situ ITA with a subsidiary one. There is a paucity of research in the comparison of outcomes between the I- and Y-grafts [18]. Nakajima et al. [19] reported that the composite Y-graft configuration had lower pressure capacity than the straight graft, despite increased flow capacity. The functional adequacy of a bypass conduit is determined by flow capacity and pressure capacity. The pressure capacity of the conduit may be determined using certain anatomic characteristics, such as its shape and length. The impact of these two factors is more influential in multi-vessel disease, which requires a greater number of distal anastomoses. A previous study has also shown the advantage of the Y-graft, as the rapid growth of the in situ ITA composite graft increases the amount of graft flow [20]. The contralateral selection of the in situ ITA graft to the AVF is easy to accompany with anatomically unfavorable anastomosis caused by the biased effort to avoid using an in situ ITA on the ipsilateral side of an AVF. The simplest graft configuration, determined using individual patients’ coronary artery anatomy, should be preferentially selected. Using the ITA as a free graft from the aorta could represent a solution to these challenges [21,22]. However, severe atherosclerotic change of the ascending aorta is a frequent finding in dialysis-dependent patients, making it unfeasible for the ITA to be used as a free graft from the ascending aorta [23]. A bypass strategy of using bilateral in situ ITA can be an alternative option. However, the use of bilateral ITAs in dialysis-dependent patients present concerns about postoperative sternal wound problems. Optimal bypass graft configuration is not well established for dialysis-dependent patients.

The baseline characteristics, rather than the ipsilateral location of the AVF, may affect the late outcomes more significantly. The previous study identified age >63 years, DM and PAOD as predictors for late death and DM, and left ventricular ejection fraction <0.40 as predictors for late cardiac events after CABG in hemodialysis patients [24]. Kai et al. [25] identified calcification of the aorta, peripheral vascular disease, insulin-dependent diabetes and age as independent risk factors for late death in bilateral ITA grafting in dialysis-dependent patients. The results of risk factor analysis of late cardiac events from our study were similar to those of the previous studies: left ventricular dysfunction, myocardial infarction, and PAOD were statistically significant risk factors.

There are several limitations to this study. First, this study was a retrospective observational study, therefore the exact number of coronary steals could not be determined. Accordingly, we investigated recurrent angina and coronary re-intervention as an alternative endpoint to coronary steal. Second, the number of patients enrolled was relatively small and the baseline patient characteristics showed difference between the groups. Thus, we applied IPW adjustment to make balance between the two groups. Third, coronary angiography during the follow-ups was not performed in all patients, limiting the evaluation of coronary steal.

## 5. Conclusions

CABG using an in situ ITA on the ipsilateral side of an upper-arm AVF as a blood source (graft inflow) did not deteriorate the late cardiac outcomes compared with bypass grafting using an in situ ITA on the contralateral side, in terms of MACCE and the composite outcome of recurrent angina and coronary re-intervention. Moreover, with susceptibility of coronary steal phenomenon, it was not an independent risk factor for late cardiac events. Additionally, the baseline patient characteristics and composite graft configuration were significant predictors for the late adverse cardiac events. The poor outcomes may result from the unfavorable anastomosis caused by the predisposition to avoid using an in situ ITA ipsilateral to AVF. Therefore, it might not be necessary to avoid using an in situ ITA on the ipsilateral side of an upper-arm AVF for optimal coronary artery bypass grafting in dialysis dependent patients.

## Figures and Tables

**Table 1:** Patient characteristics.

	Ipsilateral (*n* = 22)	Contralateral (*n* = 21)	*p*-Value	Ipsilateral (IPW Cohort)	Contralateral (IPW Cohort)	*p*-Value
Age, y	63.0 ± 8.1	63.1 ± 9.1	0.865	60.2 ± 7.4	62.3 ± 8.5	0.469
Male, *n* (%)	17 (77.3)	15 (71.4)	0.661	84.0	74.6	0.495
Body mass index	23.8 ± 3.2	23.2 ± 4.7	0.477	24.5 ± 2.6	23.6 ± 5.3	0.587
Hypertension, *n* (%)	18 (81.8)	18 (85.7)	>0.999	97.5	93.5	0.447
Smoking, *n* (%)	2 (9.1)	1 (4.8)	>0.999	9.0	11.0	0.870
Dyslipidemia, *n* (%)	5 (22.7)	6 (28.6)	0.661	16.5	24.5	0.567
DM, *n* (%)	18 (81.8)	17 (80.9)	>0.999	89.1	83.2	0.583
Stroke history, *n* (%)	6 (27.3)	6 (28.6)	0.924	19.7	30.3	0.508
COPD, *n* (%)	2 (9.1)	3 (14.3)	0.664	7.7	15.7	0.468
PAOD, *n* (%)	4 (18.2)	10 (47.6)	0.039	46.6	39.7	0.773
Atrial fibrillation, *n* (%)	4 (18.2)	1 (4.8)	0.370	9.8	4.9	0.550
Porcelain aorta, *n* (%)	3 (13.6)	6 (28.6)	0.407	12.0	22.7	0.414
MI history, *n* (%)	6 (27.3)	8 (38.1)	0.666	49.4	35.3	0.541
PCI history, *n* (%)	7 (31.8)	8 (38.1)	0.911	54.6	39.2	0.488
Acute MI, *n* (%)	7 (31.8)	9 (42.9)	0.665	21.9	37.7	0.339
LV dysfunction, *n* (%)	5 (22.7)	6 (28.6)	0.736	16.5	27.3	0.458
3VD, *n* (%)	14 (63.6)	20 (95.2)	0.021	81.30	92.9	0.346
LM disease, *n* (%)	6 (27.3)	6 (28.6)	0.924	18.0	23.7	0.678
Side of AVF, *n* (%)						
Right arm	0	5 (23.8)				
Left arm	22 (100)	16 (76.2)				

IPW, inverse probability weighting; DM, diabetes mellitus; COPD, chronic obstructive pulmonary disease; PAOD, peripheral arterial occlusive disease, MI, myocardial infarction; PCI, percutaneous coronary intervention; LV, left ventricular; 3VD, three-vessel disease; LM, left main; AVF, arteriovenous fistula.

**Table 2:** Operative data.

	Ipsilateral (*n* = 22)	Contralateral (*n* = 21)	*p*-Value
Emergency, *n* (%)	1 (4.5)	2 (9.5)	0.607
Isolated CABG, *n* (%)	17 (77.3)	16 (76.2)	>0.999
Concomitant surgery, *n* (%)	5 (22.7)	5 (23.8)	>0.999
CABG type			
Conventional, *n* (%)	9 (40.9)	7 (33.3)	0.607
On-pump beating, *n* (%)	3 (13.6)	4 (19.0)	0.691
Off-pump, *n* (%)	10 (45.5)	10 (47.6)	0.887
Complete revascularization, *n* (%)	15 (68.2)	16 (76.2)	0.343
Number of distal anastomosis	2.7 ± 1.4	3.1 ± 0.8	0.361
Distal anastomosis			
LAD territory, *n* (%)	22 (100.0)	20 (95.2)	0.300
LCX territory, *n* (%)	15 (68.2)	17 (81.0)	0.488
RCA territory, *n* (%)	12 (54.5)	19 (90.5)	0.016
Graft configuration			
In situ LITA inflow, *n* (%)	21 (95.5)	5 (23.8)	<0.001
In situ RITA inflow, *n* (%)	1 (4.5)	16 (76.2)	<0.001
In situ LITA to LAD, *n* (%)	21 (95.5)	5 (23.8)	<0.001
Y anastomosis, *n* (%)	10 (45.5)	7 (33.3)	0.537
I anastomosis, *n* (%)	1 (4.5)	14 (66.7)	<0.001
Selection of conduits			
Additional SVG use, *n* (%)	6 (27.3)	7 (33.3)	0.747
Total arterial graft, *n* (%)	16 (72.7)	14 (66.7)	0.665

CABG, Coronary artery bypass grafting; LAD, left anterior descending artery; LCX, left circumflex artery; RCA, right coronary artery; LITA, left internal thoracic artery; RITA, right internal thoracic artery; BITA, bilateral internal thoracic artery; SVG, saphenous vein graft.

**Table 3:** Early outcomes.

	Ipsilateral (*n* = 22)	Contralateral (*n* = 21)	*p*-Value	Ipsilateral (IPW Cohort)	Contralateral (IPW Cohort)	*p*-Value
Hospital death	0	4 (19.0)	0.104	0	24.9	0.018
Stroke	0	1 (4.8)	0.981	0	3.0	0.260
Atrial fibrillation	3 (13.6)	2 (9.5)	>0.999	7.0	10.5	0.685
Respiratory problem	3 (13.6)	1 (4.8)	0.634	7.5	9.5	0.838
Mediastinitis	0	1 (4.8)	>0.999	0	3.2	0.264
Bleeding reoperation	1 (4.5)	3 (14.3)	0.566	2.5	9.8	0.235

**Table 4:** Risk factor analysis (unadjusted cohort).

MACCE	Multivariable Analysis	
	Hazard Ratio [95% CI]	*p*-Value
Insulin-dependent DM	-	0.410
COPD	-	0.495
Left ventricular dysfunction	5.180 [1.864–14.394]	0.002
I anastomosis	-	0.147
PAOD	4.185 [1.349–12.979]	0.013
Ipsilateral location	-	0.203
**Recurrent Angina and Reintervention**	**Multivariable Analysis**	
	**Hazard Ratio [95% CI]**	***p*** **-Value**
Acute myocardial infarction	3.705 [1.207–11.374]	0.022
Old myocardial infarction	3.617 [1.064–12.302]	0.040
I anastomosis	5.169 [1.342–19.907]	0.017
Ipsilateral location	-	0.533

DM, diabetes mellitus; COPD, chronic obstructive pulmonary disease; PAOD, peripheral arterial occlusive disease; LAD, left anterior descending artery; LCX, left circumflex artery.

**Table 5:** Risk factor analysis (inverse probability weighting-adjusted cohort).

MACCE	Multivariable Analysis	
	Hazard Ratio [95% CI]	*p*-Value
COPD	5.667 [1.599–20.088]	0.007
Left ventricular dysfunction	4.016 [1.419–11.358]	0.009
I anastomosis	2.859 [1.080–7.565]	0.034
Ipsilateral location	-	0.145
**Recurrent Angina and Reintervention**	**Multivariable Analysis**	
	**Hazard Ratio [95% CI]**	***p*** **-Value**
Old myocardial infarction	4.489 [1.241–16.234]	0.022
History of PCI	-	0.319
Complete revascularization	-	0.520
Y anastomosis	-	0.365
I anastomosis	8.817 [1.859–41.809]	0.006
Ipsilateral location	-	0.824

COPD, chronic obstructive pulmonary disease; LAD, left anterior descending artery; PCI, percutaneous coronary intervention; LCX, left circumflex artery.

## Data Availability

The data presented in this study are available on request from the corresponding author.
